# Safety of Percutaneous Dilatational Tracheostomy Under Uninterrupted Therapeutic Anticoagulation

**DOI:** 10.3390/jcm15134877

**Published:** 2026-06-23

**Authors:** Bernhard Zapletal, Marcus J. Schultz, Michael J. Brenner, Severin Laengle, Edda M. Tschernko

**Affiliations:** 1 Department of Anesthesiology, General Intensive Care and Pain Medicine, Division of Cardiac Thoracic Vascular Anesthesia and Intensive Care Medicine, Medical University Vienna, 1090 Vienna, Austria; 2 Department of Anaesthesiology, Rescue- and Pain Medicine, Cantonal Hospital St. Gallen, HOCH Health Ostschweiz, 9007 St. Gallen, Switzerland; 3 Department of Otolaryngology–Head & Neck Surgery, University of Michigan Medical Center, Ann Arbor, MI 48109, USA; 4 Department of Cardiac and Thoracic Aortic Surgery, Medical University Vienna, 1090 Vienna, Austria

**Keywords:** tracheostomy, LVAD, ECMO, anticoagulation, bleeding

## Abstract

**Background**: Percutaneous dilatational tracheostomy (PDT) is increasingly performed without interrupting therapeutic anticoagulation in critically ill patients with extracorporeal membrane oxygenation (ECMO) or ventricular assist devices (VADs). However, the safety of PDT performed under ongoing therapeutic anticoagulation, particularly regarding periprocedural bleeding risk, remains uncertain. This study compared periprocedural bleeding complications between patients undergoing PDT under therapeutic and prophylactic anticoagulation. **Methods**: This observational cohort study in a cardiovascular ICU included all patients who underwent PDT between 2016 and 2024. The cohort comprised critically ill patients receiving uninterrupted therapeutic anticoagulation for ECMO, VAD, MVs (mechanical heart valves), and arrhythmia, as well as patients receiving low-dose anticoagulation for venous thromboprophylaxis. The primary endpoint was any severe procedure-related or late bleeding complication, while secondary endpoints included all minor procedure-related or late bleeding complications. **Results**: The cohort included 174 patients of whom 84 (48.3%) underwent PDT receiving uninterrupted therapeutic anticoagulation for ECMO, VAD, MVs, or arrhythmia. None experienced severe procedure-related bleeding. The incidence of major and minor bleeding complications did not differ between patients receiving uninterrupted therapeutic anticoagulation and those undergoing PDT under low-dose prophylactic anticoagulation. Other bleeding complications were also rare and comparable between the two groups. **Conclusions**: In this cohort, the incidence of severe and minor bleeding was low among patients undergoing PDT under uninterrupted therapeutic anticoagulation for ECMO, VAD, MVs, or arrhythmia and did not differ from that in patients receiving low-dose anticoagulation for venous thromboprophylaxis. BMI, but not anticoagulation intensity, was independently associated with post-PDT bleeding.

## 1. Introduction

Percutaneous dilatational tracheostomy (PDT) has become widely used in intensive care medicine to facilitate ventilator weaning, shorten intensive care unit (ICU) stay, reduce ventilation-associated complications, lower sedation requirements, and improve clearance of pulmonary secretions [1,2,3]. The procedure is considered safe, with severe life-threatening early complications mainly due to bleeding occurring in less than 5% of cases [4,5,6]. Percutaneous dilatational tracheostomy (PDT) is increasingly performed in complex patients [6,7,8,9], particularly those requiring continuous anticoagulation for extracorporeal membrane oxygenation (ECMO) and ventricular assist devices (VAD), where uninterrupted therapeutic anticoagulation is critical to prevent thrombosis within extracorporeal circuits [5]. The challenge encountered in patients with mechanical circulatory support (MCS) extends to patients receiving anticoagulation for mechanical heart valves (MVs), significant arrhythmias, or major thrombosis requiring therapeutic anticoagulation.

PDT is usually performed after brief interruptions of anticoagulation before the procedure [5,10]. However, clinicians must balance the procedural bleeding risk of continuing therapeutic anticoagulation against the thromboembolic risk of interruption. In the absence of a clear consensus regarding the handling of anticoagulation during PDT, clinical practice varies widely between institutions. Consequently, PDT may be deferred or avoided in patients perceived to be at elevated bleeding risk, with some centers favoring surgical tracheostomy, despite broadly similar rates of major complications between techniques [11,12,13].

Ethical limitations restrict the conduct of randomized controlled trials comparing suspended with uninterrupted anticoagulation during PDT in the above-described patient collective. Therefore, it remains unclear whether suspended anticoagulation with, e.g., low-molecular-weight heparin (LMWH) or continuous argatroban infusions is safe during PDT. To the best of our knowledge, only one study included patients with uninterrupted intravenous unfractionated heparin (UFH). Moreover, previous investigations have largely been descriptive and have not directly compared patients receiving therapeutic anticoagulation with those receiving standard prophylactic anticoagulation [5,9]. Consequently, the incremental bleeding risk attributable to uninterrupted therapeutic anticoagulation during PDT remains unclear, particularly in patients requiring continuous anticoagulation because of ECMO, VADs, MVs, or other high-risk thrombotic conditions.

Given the limited evidence regarding the safety of PDT under high-dose uninterrupted therapeutic anticoagulation, particularly in patients requiring anticoagulation for ECMO, VAD, or MVs, the primary aim of this study was to compare periprocedural bleeding complications in critically ill patients undergoing PDT while receiving high-dose uninterrupted therapeutic anticoagulation versus low-dose anticoagulation for DVT prophylaxis. We hypothesized that uninterrupted therapeutic anticoagulation would not be associated with an increase in bleeding complications.

## 2. Materials and Methods

### 2.1. Design

This is a retrospective observational study including patients who underwent PDT between October 2016 and May 2024 in the cardiovascular ICU of a tertiary university hospital in Vienna, Austria, at the University Hospital Vienna. The study was approved by the Institutional Review Board (EC number 1736/2024) and conducted in accordance with the Declaration of Helsinki. The need for individual patient consent was waived by the IRB due to the retrospective nature of the investigation.

### 2.2. Patients

Patients were eligible if (i) aged 18 years or older; (ii) admitted to the cardiovascular ICU during the study period; and (iii) underwent tracheostomy under anticoagulation. Patients who underwent (a) surgical tracheostomy, (b) tracheostomy before admission to our ICU, or (c) had previously undergone tracheostomy were excluded. Patients (d) who remained in the ICU for less than 7 days after PDT were also excluded, to ensure a complete follow-up period for late bleeding complications.

### 2.3. PDT Procedure and Personnel

In our ICU, PDT is typically performed in deeply sedated, intubated patients under controlled ventilation, with muscle relaxation when required. After positioning, the planned insertion site is routinely screened by ultrasound. All procedures are performed under bronchoscopy guidance. Preprocedural assessment included laryngeal handshake and ultrasound in all patients. A linear-array ultrasound transducer (13–6 MHz; HFL38xi, Sonosite, Bothell, WA, USA) was used for preprocedural evaluation to identify relevant pre-tracheal vascular structures and, when indicated, for real-time procedural guidance. The cricoid cartilage is identified, and a tracheostomy location is marked 2 to 3 tracheal rings below the cricoid cartilage. PDT is undertaken only in the absence of large, poorly compressible venous structures or relevant arterial anatomic variants at the planned insertion site. When such vascular findings are present, surgical tracheostomy is pursued if deemed feasible by an otolaryngology specialist; if not feasible, tracheostomy is deferred. Indications for additional real-time ultrasound guidance during the procedure included a body mass index (BMI) > 35 kg/m^2^, inability to reliably localize the trachea by laryngeal handshake, or significant cervical and thoracic scoliosis. Under sterile conditions and bronchoscopic guidance, a guidewire and dilator are introduced prior to insertion of the tracheal cannula. Either the PercuTwist (Rüsch, Kernen, Germany) or Tracoe^®^ Percutan tracheostomy sets (Tracoe^®^ medical GmBH, Frankfurt, Germany) is used according to consultant preference. Bilateral backstitch sutures adjacent to the insertion site were placed to limit local bleeding. Indications included a BMI > 35 kg/m^2^ or intraoperative bleeding during skin incision or dilatation to reduce the risk of postprocedural bleeding.

At the beginning of the study period, PDT was performed by a limited number of physicians with extensive prior experience (>50 procedures) and exposure to various tracheostomy systems. Over time, procedural expertise was disseminated among interested ICU consultants. To minimize the risk of complications, all PDT procedures were conducted by two physicians, one performing bronchoscopy and the other tracheostomy, with an experienced senior consultant acting either as operator or supervisor.

### 2.4. Anticoagulation Management

In our ICU, patients requiring therapeutic anticoagulation for MCS, ECMO, or MVs are usually treated with LMWH twice daily, or with fondaparinux in case of heparin-induced thrombocytopenia (HIT). Anti-factor Xa activity is assessed, targeting a trough anti-factor Xa activity level of 0.3 to 0.5 IU/L. In patients receiving continuous intravenous unfractionated heparin (UFH), an anti-factor Xa activity level of 0.2 to 0.5 IU/L and an activated prothrombin time (aPTT) of 1.5 times the upper limit of normal (55 to 65 s) is targeted. In patients treated with argatroban, a hemoclot assay of 0.2 to 0.5 and an aPTT of 55 to 65 s is targeted.

Patients receiving anticoagulation for venous thromboprophylaxis usually receive LMWH once daily, with the dose depending on patient weight, renal function, and known coagulation deficits. In these patients, a trough anti-factor Xa activity level of <0.1 to 0.1 is targeted to avoid accumulation.

After PDT, anticoagulation was continued in all patients, except in the absence of acute bleeding.

### 2.5. Data Collected

We collected demographic and baseline data for each patient, including sex, age, BMI, ICU admission diagnosis, the Simplified Acute Physiology Score (SAPS) 3 at ICU admission, and the Sequential Organ Failure Assessment (SOFA) score on the day of PDT. We recorded anticoagulation status and indication, indication for PDT, days of intubation before PDT, FiO_2_ before PDT, and the size of the first tracheostomy cannula used. We also collected time to first cannula exchange, duration of respiratory weaning until decannulation, and 30-day mortality after PDT.

Anticoagulation details included anti-Xa activity levels (in patients receiving LMWH, fondaparinux, and UFH), hemoclot assay (in patients receiving argatroban), aPTT (in patients receiving UFH and argatroban), INR (in patients receiving phenprocoumon), and platelet count. Additionally, the presence of antiplatelet therapy at the time of PDT was assessed.

Baseline blood hemoglobin, INR, aPTT, fibrinogen, and platelet count were documented. Transfused blood products and coagulation factors were recorded from 48 h before to 48 h after PDT. Bleeding disorders known before PDT were noted.

### 2.6. Definitions

Severe bleeding complications were classified as procedure-related (occurring during or within 48 h after PDT) or late (occurring >48 h to 7 days after PDT) and were defined as bleeding uncontrolled by compression or topical hemostatic measures, or as intratracheal bleeding unresponsive to cold saline instillation or local vasopressors. All other bleeding complications were defined as minor and were also classified as procedure-related or late.

Severe non-bleeding complications were classified as procedure-related if life-threatening hemodynamic (severe hypotension, i.e., mean arterial blood pressure < 50 mmHg) or respiratory complications (severe hypoxia, i.e., SpO_2_ < 80% or PaO_2_ < 50mmHg, or hypercapnia, with PaCO_2_ > 70 mmHg), posterior wall injury, false passage, pneumothorax, failed procedure, or conversion to surgical tracheostomy. Late non-bleeding complications included cannula obstruction or dislocation, localized infection, or sepsis originating from the tracheostomy site.

### 2.7. Study Endpoints

The primary endpoint was a composite of any severe bleeding complication. Secondary endpoints included all minor bleeding and any severe and minor non-bleeding complications.

### 2.8. Statistical Analysis

The number of patients who underwent PDT in the cardiovascular ICU during the study period served as the sample size. Data are presented as absolute counts and proportions, means and standard deviations (SD), or medians and interquartile ranges (IQR), depending on data distribution, where appropriate.

Severe bleeding complications, minor bleeding complications, and non-bleeding complications, reported as counts and proportions, are compared between patients with uninterrupted therapeutic anticoagulation for ECLS, MCS, or MVs to those receiving anticoagulation for venous thromboprophylaxis using X^2^-tests and unpaired *t*-tests as appropriate.

In patients with uninterrupted therapeutic anticoagulation for ECLS, MCS, or MVs, we also report severe bleeding complications for patients managed with LMWH, fondaparinux, UFH, argatroban, or phenprocoumon.

To identify independent predictors of bleeding after PDT, a post hoc multivariable logistic regression analysis was conducted. The model included age, sex, BMI, therapeutic anticoagulation status, coagulation parameters, and PDT performed during ECMO or VAD support as covariates.

All statistical analyses were conducted using SPSS for Windows, version 29.0 (IBM Corp., Armonk, NY, USA). A *p*-value < 0.05 was considered statistically significant.

## 3. Results

### 3.1. Patients

**Figure 1:**
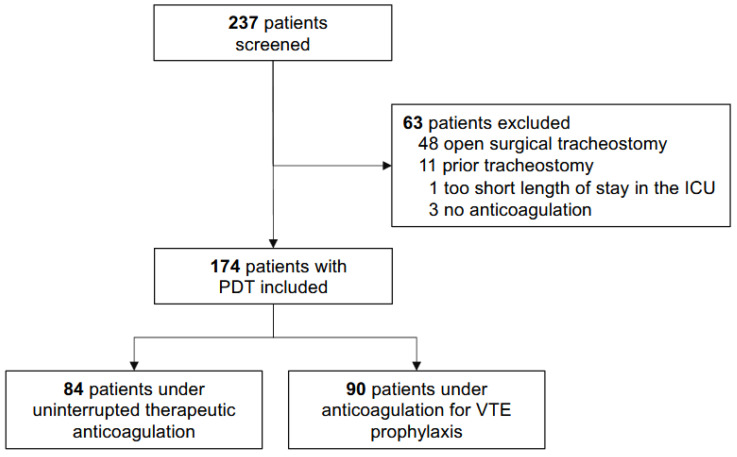
Flowchart of patients. Abbreviations: ICU, intensive care unit; PDT, percutaneous dilatational tracheostomy.

Of 237 patients who underwent tracheostomy during the study period, 60 were excluded. The main reasons for exclusion were open surgical tracheostomy and prior tracheostomy (Figure 1).

Among the remaining 174 patients, 84 (48.3%) underwent PDT under uninterrupted therapeutic anticoagulation for MCS, ELS, or MVs, while 90 (51.7%) underwent PDT under anticoagulation for venous thromboprophylaxis. Patients undergoing PDT under uninterrupted therapeutic anticoagulation were younger and more often male. Patients under full therapeutic anticoagulation also suffered more frequently from heparin-induced coagulopathy (HIT) and had undergone reintubation, while PDT in patients under anticoagulation for DVT prophylaxis was more frequently performed for neurological reasons. Transfusion of packed red cells, fresh frozen plasma, platelet concentrates, fibrinogen concentrates, and prothrombin complex concentrates did not differ between groups (Table 1).

Table 2 summarizes anticoagulation regimens and laboratory measures of anticoagulation intensity at the time of PDT. Fewer patients in the therapeutic anticoagulation group received LMWH due to more frequent administration of UFH or argatroban. Patients under therapeutic anticoagulation received more intensive anticoagulation, reflected by higher mean anti-Xa levels, compared to patients receiving anticoagulation for venous thromboprophylaxis. Anticoagulation monitoring parameters, including anti-Xa activity, aPTT, and hemoclot assay results, were within the intended therapeutic or prophylactic target ranges in most patients, supporting adequate separation between the study groups with respect to anticoagulation intensity.

### 3.2. Severe Bleeding Complications

The primary composite endpoint of early severe bleeding complications neither occurred in patients under uninterrupted therapeutic anticoagulation for MCS, ELS, or MVs, nor in patients undergoing PDT under anticoagulation for venous thromboprophylaxis (Table 3).

### 3.3. Mild Bleeding Complications

Procedure-related mild bleeding complications occurred in six (7.1%) patients under therapeutic anticoagulation compared to five (5.6%) patients under anticoagulation for venous thromboprophylaxis (*p* = 0.67, Table 3); five under LMWH and one under argatroban. Late mild bleeding complications neither occurred in patients under therapeutic anticoagulation nor in patients under anticoagulation for venous thromboprophylaxis (Table 3). Rates of bleeding complications were not different between patients receiving either LMWH, fondaparinux, UFH, argatroban, or phenprocoumon for uninterrupted therapeutic anticoagulation (*p* = 0.87).

### 3.4. Other Complications

Severe procedure-related complications were rare and occurred slightly more often in patients receiving anticoagulation for DVT prophylaxis than in those under uninterrupted therapeutic anticoagulation (Table 3). Severe late complications were absent in patients receiving therapeutic anticoagulation and occurred in only a single patient in the prophylactic anticoagulation group. Minor early and late procedure-related complications were uncommon and occurred at similar rates in both groups.

### 3.5. Post Hoc Analysis

In post hoc multivariable logistic regression analysis, BMI was the sole independent predictor of bleeding after PDT (Table 4).

## 4. Discussion

This study compared the occurrence of severe bleeding during or after PDT between patients under therapeutic anticoagulation for EMCO, VADs, MVs, arrhythmia, significant thrombosis, and those receiving low-dose anticoagulation for DVT prophylaxis. The key findings are: (i) no severe PDT-related bleeding complications occurred in either group; (ii) minor bleeding complications, both early and late, were rare and occurred at similar rates in the two groups; and (iii) other complications were uncommon and similarly distributed between groups.

The study has several strengths. A substantial number of patients undergoing PDT under uninterrupted therapeutic anticoagulation were included and compared with those receiving anticoagulation for DVT prophylaxis. Anticoagulation targets were achieved in the vast majority of patients prior to PDT. Consecutive patients were included over a predefined time period, with minimal exclusion criteria limited to surgical tracheostomy, prior tracheostomy, or insufficiently long follow-up to capture post-procedural complications. PDT procedures and early and late complications were systematically documented daily by the intensivists and nursing staff who were unaware of the study, minimizing reporting (capture) bias. The single-center setting ensured standardized procedural and anticoagulation management, and the inclusion of diverse anticoagulation regimens enhances generalizability across different clinical scenarios. Finally, a prespecified analysis plan was strictly followed.

The present findings are broadly consistent with the existing literature, which reports a low incidence of severe bleeding complications after PDT in critically ill patients [5,14]. Large series have shown that major bleeding is uncommon [14] and that most patients experience none or only mild bleeding after PDT, even in cohorts with coagulopathy or risk factors for bleeding [5,7,8,9]. Meta-analytic data also suggest that anticoagulation itself is not significantly associated with increased intraoperative bleeding compared with no anticoagulation [15], although studies vary in definitions, management strategies, and cessation periods of anticoagulation before PDT. Smaller retrospective studies including patients on therapeutic levels of anticoagulation have reported complication rates [16,17] comparable to those observed in cohorts without ongoing anticoagulation [8,9]. In line with these data, the present study extends the current evidence by showing that severe bleeding is rare, even in patients undergoing PDT while receiving uninterrupted therapeutic doses of LMWH and argatroban.

To date, no previous study has included patients receiving therapeutic doses of LMWH or argatroban, and to directly compare patients under uninterrupted therapeutic anticoagulation with those receiving LMWH for venous thromboprophylaxis, with anti-Xa levels reported in both groups. The incidence of minor bleeding complications was lower than in previous reports [5,7,8], which may partly reflect differences in the definitions of such events. Another contributing factor that may explain this difference, particularly minor bleeding complications, may be the practice of placing backstitch sutures adjacent to the PDT site immediately after the procedure. This approach, not described in prior studies, may reduce the need for local compression or topical hemostatic agents to control minor bleeding.

No severe bleeding occurred in either group, and the incidence of minor bleeding was not higher among patients receiving uninterrupted therapeutic anticoagulation. Post hoc multivariable logistic regression identified BMI as the sole independent predictor of bleeding after PDT, irrespective of anticoagulation status. These results indicate that PDT can be performed under therapeutic anticoagulation, including LMWH and argatroban, without an apparent increase in clinically relevant bleeding. This has practical implications for ICU workflow, as PDT may not need to be delayed solely to allow anticoagulant effects to subside. Avoiding such delays could simplify anticoagulation management, facilitate ICU logistics, and reduce complications associated with prolonged endotracheal intubation [3]. Because interruption of anticoagulation in this population carries a risk of adverse events, including circuit and pump thrombosis in patients with MCS and thromboembolic events in patients with mechanical valves, maintaining anticoagulation rather than routinely interrupting it before PDT or postponing the procedure until coagulation normalizes may represent a clinically sound strategy [7,8,11,18,19]. In this context, the present results further indicate that the presence of anticoagulation alone need not automatically prompt the necessity of an open surgical approach, provided that patient selection is appropriate and the procedure is performed by experienced operators. Nevertheless, PDT-related bleeding can still occur, underscoring the importance of operator experience, standardized protocols, adjunctive use of ultrasound in addition to bronchoscopy guidance in selective cases, and measures such as peri-cannular sutures. In patients with external surgical bleeding, such sutures are immediately placed if not already performed. While external bleeding can usually be managed with local hemostatic measures, endobronchial bleeding is considered a life-threatening condition, irrespective of whether it results from endotracheal bleeding after PDT, instrumentation with guidewires, suction maneuvers, or ECMO- or VAD-associated coagulopathy. Initial management in our center consists of local hemostatic measures via flexible bronchoscopy, including topical vasopressors and hemostatic agents, and if required, hemostatic swabs applied using forceps [20,21,22,23].

After control of acute bleeding, cryoextraction by bronchoscopy and endoscopic forceps are used to remove obstructing blood clots [24] while rigid bronchoscopy, endovascular coiling, or surgical intervention are reserved for severe or refractory cases [25].

In patients under therapeutic anticoagulation, treatment is reduced to prophylactic dosing in the event of bleeding after PDT, whereas prophylactic anticoagulation is temporarily discontinued until resolution. Administration of coagulation factor concentrates in cases of surgical bleeding is avoided in patients under therapeutic anticoagulation, particularly in patients under ECMO or VAD due to potentially dire thrombotic complications [26,27].

### 4.1. Study Limitations

The retrospective, single-center design limits causal inference and generalizability, and baseline differences between groups may have introduced residual confounding. The retrospective design limited nuances regarding the characterization of bleeding severity. Consequently, clinically relevant differences within the predefined categories of minor and severe bleeding may not have been fully captured. Anticoagulation was not uniform but varied by indication and agent, which may have influenced bleeding risk [28], although targets were guided by established recommendations [29,30,31]. A true non-anticoagulated control group was not available, as PDT without at least prophylactic anticoagulation is uncommon and generally limited to patients with active bleeding or severe coagulopathy. Consequently, only three of 237 screened patients met these criteria (Figure 1). Nevertheless, the use of a prophylactic anticoagulation group as a comparator may enhance the clinical relevance of the findings, as routine thromboprophylaxis represents standard care for most critically ill patients. The use of two different PDT systems (PercuTwist and Tracoe^®^ Percutan) represents an additional limitation. Incomplete documentation resulting from the retrospective design precluded analysis of the relationship between device type and bleeding complications. However, previous studies have reported similar complication rates across PDT techniques, and operator experience and device familiarity may be more relevant determinants of outcome than the choice of device itself [32,33,34]. Complications were systematically recorded, and patients were observed for at least 7 days, but later outcomes such as tracheal stenosis or swallowing dysfunction were not assessed. The requirement for a minimum ICU stay of 7 days after PDT may have led to the exclusion of patients with unrecognized complications. However, only one patient was excluded for this reason, and retrospective review of the medical records identified no procedure-related complications. Anti-Xa or aPTT measurements immediately before PDT were unavailable in 10 patients (6%) because of sample processing or analytical issues (hemolysis and interference of high plasma bilirubin). However, manual review of the medical records confirmed correct classification of all patients into the therapeutic or prophylactic anticoagulation groups. Finally, these findings cannot be extrapolated to surgical tracheostomy or to patients receiving direct oral anticoagulants or vitamin K antagonists, which were not represented in this cohort.

### 4.2. Recommendations for Further Research

While ethical limitations restrict the conduct of randomized controlled trials, future prospective multicenter studies are needed to validate these findings in larger patient populations and across different institutions. Standardized definitions and prospective recording of bleeding complications and assessment of anticoagulation intensity using various anticoagulants may help further characterize the procedural risks associated with PDT under uninterrupted high-dose anticoagulation.

## 5. Conclusions

PDT performed under ongoing therapeutic anticoagulation was not associated with a higher incidence of bleeding complications compared with PDT performed under anticoagulation for venous thromboprophylaxis. In post hoc multivariable analysis, BMI was the sole independent predictor of bleeding after PDT. The present results support the view that routine interruption of anticoagulation solely to perform PDT may not be necessary in selected patients.

## Figures and Tables

**Table 1:** Patient demographics, baseline characteristics, and PDT details.

	Patients Under Therapeutic Anticoagulation N = 84	Patients Under Anticoagulation for DVT Prophylaxis N = 90	*p*
age, years	61.5 (12.6)	65.5 (11.3)	0.03
gender, female	18 (22.5)	34 (38.6)	0.02
BMI, kg/m^2^	27.6 (6.1)	26.1 (6.1)	0.15
SAPS 3 score	60.1 (13.4)	60.7 (15.8)	0.80
ICU admission diagnosis, n (%)			0.38
cardiomyopathy	20 (23.8)	12 (13.3)	
aortic dissection	11 (13.1)	15 (16.1)	
cardiac valve disease	17 (20.2)	19 (21.1)	
endocarditis	7 (8.3)	8 (8.6)	
coronary heart disease/ACS	7 (8.3)	11 (11.8)	
pneumonia/sepsis	7 (8.4)	4 (4.4)	
aortic aneurysm	3 (3.6)	6 (6.5)	
CPR	2 (2.4)	3 (3.2)	
cardiogenic shock	4 (4.8)	1 (1.1)	
HOCM + CAD	0 (0.0)	2 (2.2)	
other	6 (7.1)	9 (10.0)	
indication for full anticoagulation, n (%)			–
MCS	32 (38.1)		
mechanical valves	9 (10.7)		
atrial fibrillation/arrhythmias	20 (23.8)		
venous thrombus	8 (9.5)		
arterial/intracardiac thrombosis	7 (8.3)		
pulmonary embolism	3 (3.6)		
complex endovascular stent graft	5 (6.0)		
indication for PDT, n (%)			0.03
prolonged ventilation	28 (33.3)	33 (36.7)	
reintubation	36 (42.9)	21 (23.3)	
neurologic dysfunction	19 (22.6)	35 (38.9)	
bilateral recurrent laryngeal nerve palsy	0 (0.0)	1 (1.1)	
recurrent pharyngeal bleeding	1 (1.2)	0 (0.0)	
SOFA on the day of PDT	9.5 (3.8)	9.1 (3.3)	0.49
ventilator days before PDT, days	8.4 (8.6)	8.1 (6.6)	0.81
FiO_2_ on the day of PDT, fraction	0.42 (0.1)	0.43 (0.1)	0.32
coagulation disorder on the day of PDT, n (%)	16 (19.0)	5 (5.6)	<0.01
suspected or confirmed HIT	15	3	
liver failure	1	1	
ITP	0	1	
tracheostomy cannula size, mm	8.5 (0.6)	8.5 (0.6)	0.66
transfusion within 2 days before and after PDT			0.15
PRC	33 (39.3)	27 (29.0)	
FFPs	3 (3.6)	0 (0.0)	
PCs	1 (1.2)	0 (0.0)	
Fibrinogen concentrate	0 (0.0)	1 (1.1)	
PCC	1 (1.2)	2 (2.2)	

Data are means (SD), or numbers (%). Abbreviations: PDT, percutaneous dilatational tracheostomy; DVT, deep venous thrombosis; BMI, body mass Index; SAPS 3, Simplified Acute Physiology Score 3; ICU, intensive care unit; ACS, acute coronary syndrome; CnoPR, cardiopulmonary resuscitation; HOCM, hypertrophic obstructive cardiomyopathy; CAD, coronary artery disease; SOFA, Sequential Organ Failure Assessment; FiO_2_, fraction of inspired oxygen; HIT, heparin induced thrombocythemia; ITP, idiopathic thrombocytopenic purpura; MCS, mechanical circulatory support; PRC, packed red cells; FFPs, fresh frozen plasmas, PCs, platelet concentrates; PCC, prothrombin complex concentrates.

**Table 2:** Patients under specific anticoagulation, and laboratory test results on the day of PDT.

	Patients Under Therapeutic Anticoagulation N = 84	Patients Under Anticoagulation for DVT Prophylaxis N = 90	*p*
under LMWH, N	57	90	
anti-Xa levels, IU/L	0.36 (0.11)	0.05 (0.07)	<0.01
under UFH, N	9	0	
anti-Xa levels, IU/L	0.24 (0.14)		
aPTT, seconds	62.3 (15.3)		
under argatroban, N	14	0	
hemoclot assay, ng/mL	0.34 (0.13)		
aPTT, seconds	59.0 (7.1)		
under fondaparinux, N	3	0	
anti-Xa levels, IU/L	0.43 (0.04)		
under phenprocoumon	1	0	
INR	2.1		
under ASA or DAPT, N	53	57	
platelet counts, 10^9^/L	241.6 (177.2)	236.0 (140.0)	0.82

Data are presented as means (SD) and numbers. Abbreviations: LMWH, low-weight molecular heparin; anti-Xa, anti-factor Xa; UFH, unfractionated heparin; aPTT, Activated Partial Thromboplastin Time; INR, International Normalized Ratio; ASA, acetylsalicylic acid; DAPT, dual antiplatelet therapy.

**Table 3:** Primary and secondary outcomes.

	PDT Under Therapeutic Anticoagulation N = 84	PDT Under Anticoagulation for DVT Prophylaxis N = 90	*p*
Primary Outcome			
Severe bleeding, n (%)	0 (0)	0 (0)	–
Periprocedural, and 0 to 48 h	–	–	
>48 h	–	–	
Secondary Outcomes			
Severe complications, n (%)	2 (2.4)	4 (4.3)	0.09
Periprocedural, and 0 to 48 h			
Hypoxia	1 (1.2)	–	0.30
PDT failure	–	1 (1.1)	0.33
Cannula obstruction	1 (1.2)	2 (2.2)	0.60
>48 h			
Cannula obstruction	–	1 (1.1)	0.34
Minor complications, n (%)	15 (17.9)	19 (21.1)	0.61
Periprocedural, and 0 to 48 h			
Bleeding from the tracheostomy site	6 (7.1)	5 (5.6)	0.67
Hypoxia	2 (2.4)	2 (2.2)	0.94
Hypercapnia	2 (2.4)	7 (7.8)	0.11
>48 h			
Cannula dislocation	3 (3.6)	2 (2.2)	0.59
Difficult cannula exchange	2 (2.4)	3 (3.3)	0.74

Data are presented as numbers (%). For definitions of bleeding and complications, see the text. For secondary outcomes, complications are listed only if they occurred. Abbreviations: PDT, percutaneous dilatational tracheostomy.

**Table 4:** Risk factors for bleeding: multivariable logistic regression for minor bleeding.

	OR (95% CI) for Minor Bleeding	*p*
Demographic risk factors		
Age (per year)	0.97 (0.91–1.03)	0.28
Female sex (yes)	0.17 (0.17–1.75)	0.14
BMI (per point)	1.14 (1.03–1.25)	0.01
Clotting-related risk factors		
Continuous full anticoagulation (yes)	1.73 (0.27–11.07)	0.56
Normotest coagulation assay (per %)	1.00 (0.95–1.07)	0.77
INR (per point)	0.42 (0.00–138.83)	0.77
aPTT (per second)	1.00 (0.93–1.09)	0.87
Platelets (per 1000 platelets per liter)	0.31 (0.99–1.00)	0.31
aXa for LMWH or UFH (per IU per mL)	1.55 (0.36–65.75)	0.82
Fibrinogen (per mg per dL)	1.00 (0.98–1.01)	0.47
ASA (yes)	2.5 (0.26–24.02)	0.43
Other platelet function inhibitor (yes)	1.09 (0.58–2.08)	0.78
Other risk factors		
ECMO or VAD (yes)	1.36 (0.04–46.78)	0.86

Data are presented as Odds Ratio (95% CI). Abbreviations: OR, Odds Ratio; BMI, body mass index; INR, International Normalized Ratio; aPTT, Activated Partial Thromboplastin Time; aXa; factor anti-Xa; LMWH, Low-Molecular-Weight Heparin; UFH, unfractionated heparin; IU, International Units; ASA, acetylsalicylic acid; ECMO, extracorporeal membrane oxygenation; VAD, ventricular assist device.

## Data Availability

The dataset of the current study is available upon request via the corresponding author.

## References

[1] Romem A., Gilboa H. (2023). Percutaneous Tracheostomy in the ICU: A Review of the Literature and Recent Updates. Curr. Opin. Pulm. Med..

[2] Rumbak M.J., Newton M., Truncale T., Schwartz S.W., Adams J.W., Hazard P.B. (2004). A Prospective, Randomized, Study Comparing Early Percutaneous Dilational Tracheotomy to Prolonged Translaryngeal Intubation (Delayed Tracheotomy) in Critically Ill Medical Patients. Crit. Care Med..

[3] Hosokawa K., Nishimura M., Egi M., Vincent J.-L. (2015). Timing of Tracheotomy in ICU Patients: A Systematic Review of Randomized Controlled Trials. Crit. Care.

[4] Ghotbi Z., Estakhr M., Nikandish M., Nikandish R. (2023). A Modified Technique for Percutaneous Dilatational Tracheostomy. J. Intensiv. Care Med..

[5] Braune S., Kienast S., Hadem J., Wiesner O., Wichmann D., Nierhaus A., Simon M., Welte T., Kluge S. (2013). Safety of Percutaneous Dilatational Tracheostomy in Patients on Extracorporeal Lung Support. Intensiv. Care Med..

[6] Roy C.F., Silver J.A., Turkdogan S., Siafa L., Correa J.A., Kost K. (2023). Complication Rate of Percutaneous Dilatational Tracheostomy in Critically Ill Adults with Obesity. JAMA Otolaryngol. Head Neck Surg..

[7] Auzinger G., O’Callaghan G.P., Bernal W., Sizer E., Wendon J.A. (2007). Percutaneous Tracheostomy in Patients with Severe Liver Disease and a High Incidence of Refractory Coagulopathy: A Prospective Trial. Crit. Care.

[8] Kluge S., Meyer A., Kühnelt P., Baumann H.J., Kreymann G. (2004). Percutaneous Tracheostomy Is Safe in Patients with Severe Thrombocytopenia. Chest.

[9] Deppe A.-C., Kuhn E., Scherner M., Slottosch I., Liakopoulos O., Langebartels G., Choi Y.-H., Wahlers T. (2013). Coagulation Disorders Do Not Increase the Risk for Bleeding during Percutaneous Dilatational Tracheotomy. Thorac. Cardiovasc. Surg..

[10] Barton C.A., McMillian W.D., Osler T., Charash W.E., Igneri P.A., Brenny N.C., Aloi J.J., Fortune J.B. (2012). Anticoagulation Management around Percutaneous Bedside Procedures: Is Adjustment Required?. J. Trauma Acute Care Surg..

[11] Veelo D.P., Dongelmans D.A., Phoa K.N., Spronk P.E., Schultz M.J. (2007). Tracheostomy: Current Practice on Timing, Correction of Coagulation Disorders and Peri-operative Management—A Postal Survey in the Netherlands. Acta Anaesthesiol. Scand..

[12] Dempsey G.A., Morton B., Hammell C., Williams L.T., Smith C.T., Jones T. (2016). Long-Term Outcome Following Tracheostomy in Critical Care. Crit. Care Med..

[13] Freeman B.D., Isabella K., Lin N., Buchman T.G. (2000). A Meta-Analysis of Prospective Trials Comparing Percutaneous and Surgical Tracheostomy in Critically Ill Patients. Chest.

[14] Lüsebrink E., Krogmann A., Tietz F., Riebisch M., Okrojek R., Peltz F., Skurk C., Hullermann C., Sackarnd J., Wassilowsky D. (2021). Percutaneous Dilatational Tracheotomy in High-Risk ICU Patients. Ann. Intensiv. Care.

[15] Brown M., Elsawy F., Allison B., McGrath B. (2024). Antiplatelet and Anticoagulation Use and Risk of Bleeding from Percutaneous Dilatational Tracheostomy Insertion: Systematic Review and Meta-Analysis. Br. J. Anaesth..

[16] Pasin L., Frati E., Cabrini L., Landoni G., Nardelli P., Bove T., Calabrò M.G., Scandroglio A.M., Pappalardo F., Zangrillo A. (2015). Percutaneous Tracheostomy in Patients on Anticoagulants. Ann. Card. Anaesth..

[17] Takhar A., Tornari C., Amin N., Wyncoll D., Tricklebank S., Arora A., Ahmad I., Simo R., Surda P. (2020). Safety and Outcomes of Percutaneous Tracheostomy in Coronavirus Disease 2019 Pneumonitis Patients Requiring Prolonged Mechanical Ventilation. J. Laryngol. Otol..

[18] Pandian V., Vaswani R.S., Mirski M.A., Haut E., Gupta S., Bhatti N.I. (2010). Safety of Percutaneous Dilational Tracheostomy in Coagulopathic Patients. Ear Nose Throat J..

[19] Beiderlinden M., Eikermann M., Lehmann N., Adamzik M., Peters J. (2007). Risk Factors Associated with Bleeding during and after Percutaneous Dilational Tracheostomy. Anaesthesia.

[20] Peralta A.R., Chawla M., Lee R.P. (2018). Novel Bronchoscopic Management of Airway Bleeding with Absorbable Gelatin and Thrombin Slurry. J. Bronchol. Interv. Pulmonol..

[21] Jakowenko N.D., Seelhammer T.G., Nabzdyk C.G.S., Macielak R.J., Nei S.D., Kalvelage E.L., Wieruszewski P.M. (2023). Tranexamic Acid for Bleeding Management in Adult Patients on Extracorporeal Membrane Oxygenation. ASAIO J..

[22] Korraa E.E.-D.A., Madkour A.M., Galal I.H., El-Saidy I.M.I. (2017). Bronchoscopic Instillation of Tranexamic Acid to Control Bronchopulmonary Bleeding. Egypt. J. Bronchol..

[23] Valipour A., Kreuzer A., Koller H., Koessler W., Burghuber O.C. (2005). Bronchoscopy-Guided Topical Hemostatic Tamponade Therapy for the Management of Life-Threatening Hemoptysis. Chest.

[24] Tenda E.D., Yakub A., Pitoyo C.W., Fardizza F. (2016). Combination of Bronchoscopic Cryoextraction and Argon Plasma Coagulation in Treatment of Total Central Airway Obstruction Caused by Giant Blood Clot Formation in Massive Airway Bleeding. Respir. Med. Case Rep..

[25] Karmy-Jones R., Cuschieri J., Vallières E. (2001). Role of Bronchoscopy in Massive Hemoptysis. Chest Surg. Clin. N. Am..

[26] Seese L., Hickey G., Keebler M., Thoma F., Kilic A. (2020). Limited Efficacy of Thrombolytics for Pump Thrombosis in Durable Left Ventricular Assist Devices. Ann. Thorac. Surg..

[27] Farag J., Woldendorp K., McNamara N., Bannon P.G., Marasco S.F., Loforte A., Potapov E.V. (2021). Contemporary Outcomes of Continuous-Flow Biventricular Assist Devices. Ann. Cardiothorac. Surg..

[28] Alquwaizani M., Buckley L., Adams C., Fanikos J. (2013). Anticoagulants: A Review of the Pharmacology, Dosing, and Complications. Curr. Emerg. Hosp. Med. Rep..

[29] Baumgartner H., Falk V., Bax J.J., Bonis M.D., Hamm C., Holm P.J., Iung B., Lancellotti P., Lansac E., Muñoz D.R. (2017). 2017 ESC/EACTS Guidelines for the Management of Valvular Heart Disease. Eur. Heart J..

[30] Sandner S.E., Riebandt J., Haberl T., Mahr S., Rajek A., Schima H., Wieselthaler G.M., Laufer G., Zimpfer D. (2014). Low-Molecular-Weight Heparin for Anti-Coagulation after Left Ventricular Assist Device Implantation. J. Heart Lung Transplant..

[31] Shah Z., Mastoris I., Acharya P., Rali A.S., Mohammed M., Farhad S., Ranka S., Wagner S., Zanotti G., Salerno C.T. (2020). The Use of Enoxaparin as Bridge to Therapeutic INR after LVAD Implantation. J. Cardiothorac. Surg..

[32] Apfelbaum J.L., Hagberg C.A., Connis R.T., Abdelmalak B.B., Agarkar M., Dutton R.P., Fiadjoe J.E., Greif R., Klock P.A., Mercier D. (2021). 2022 American Society of Anesthesiologists Practice Guidelines for Management of the Difficult Airway. Anesthesiology.

[33] Petiot S., Guinot P.-G., Diouf M., Zogheib E., Dupont H. (2017). Learning Curve for Real-Time Ultrasound-Guided Percutaneous Tracheostomy. Anaesth. Crit. Care Pain Med..

[34] Abbott F., Ortega M., Bravo S., Basoalto R., Kattan E. (2021). Can We Improve Teaching and Learning of Percutaneous Dilatational Tracheostomy’s Bronchoscopic Guidance?. SAGE Open Med..

